# An outbreak of *Gynanisa maja* (Lepidoptera: Saturniidae) larvae in the south‐eastern lowveld of Zimbabwe

**DOI:** 10.1002/ece3.10790

**Published:** 2023-12-03

**Authors:** Allan Tarugara, Bob Mandinyenya, Bruce W. Clegg

**Affiliations:** ^1^ Malilangwe Wildlife Reserve Chiredzi Zimbabwe; ^2^ Gonarezhou National Park Chiredzi Zimbabwe

**Keywords:** caterpillar, defoliation, emperor moth, eruption, food source

## Abstract

The larvae of speckled emperor moths (*Gynanisa maja*) are important plant defoliators in savanna ecosystems of southern Africa and a valuable food resource for indigenous communities. Population explosions of *G. maja* larvae can negatively impact an area's primary productivity thereby altering herbivory patterns and associated ecosystem processes. Harvests of the larvae enhance socio‐economic livelihoods of local people by providing a source of protein and improving household incomes. We report on a population outbreak of *G. maja* larvae that occurred in south‐eastern Zimbabwe between December 2022 and January 2023 and discuss the ecological and social significance of the event. A total biomass weight of 5811 tons of *G. maja* larvae was estimated over the area of the outbreak and extensive defoliation was recorded in Colophospermum mopane trees. We could not associate the outbreak with any obvious environmental conditions and speculate that it may have been caused by subtle triggers that are not easily identified.

## Graphical abstract

This paper reports on the ecological footprint of a *Gynanisa maja* larvae outbreak that occurred between December 2022 and January 2023 in the south‐eastern lowveld of Zimbabwe.

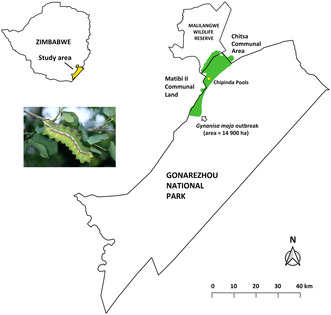

## 1 INTRODUCTION

Population explosions of insect folivores are important ecological events in savanna ecosystems (Hartnett et al., 2012). The larval (caterpillar) stage of emperor moths (family, *Saturniidae*) has both an ecological and socio‐economic significance in southern Africa (Bara et al., 2022; Ditlhogo, 1996; Thomas, 2013) with larvae of two species, the anomalous emperor (*Gonimbrasia belina* Westwood 1894) and the speckled emperor (*Gynanisa maja* Klug 1836), being important plant defoliators and a food resource for people in the region. Outbreaks of *G*. *belina* are relatively frequent and widespread (Bara et al., 2022; Straeuli, 2022), whereas those of *G*. *maja* are characterized by extended periods of relatively low incidence punctuated by population explosions that are less frequent (Singh & Satyanarayana, 2009; Thomas, 2013). Apart from incidental observation, *G. maja* has been little studied and the drivers of its eruptive dynamics are poorly understood.

Larvae of *G. maja* feed on the leaves of a wide range of savanna tree species but *Colophospermum mopane* is their most preferred food source (Chanda et al., 2022; Sileshi et al., 2007; Stone, 1991). The larvae are voracious folivores and outbreaks can defoliate large areas of woodland with implications for primary productivity, herbivory and associated cascading effects on ecosystem processes (Carson et al., 2004; Fajvan & Wood, 1996; Hartnett et al., 2012; Styles, 1994). Here we report on a *G. maja* larvae outbreak in the south‐eastern lowveld of Zimbabwe that occurred between 18 December 2022 and 6 January 2023. We aimed to (1) determine the spatial extent of the outbreak, (2) estimate the biomass of the caterpillars and (3) quantify the degree of tree and shrub defoliation, and recovery.

## 2 METHODS

**FIGURE 1:**
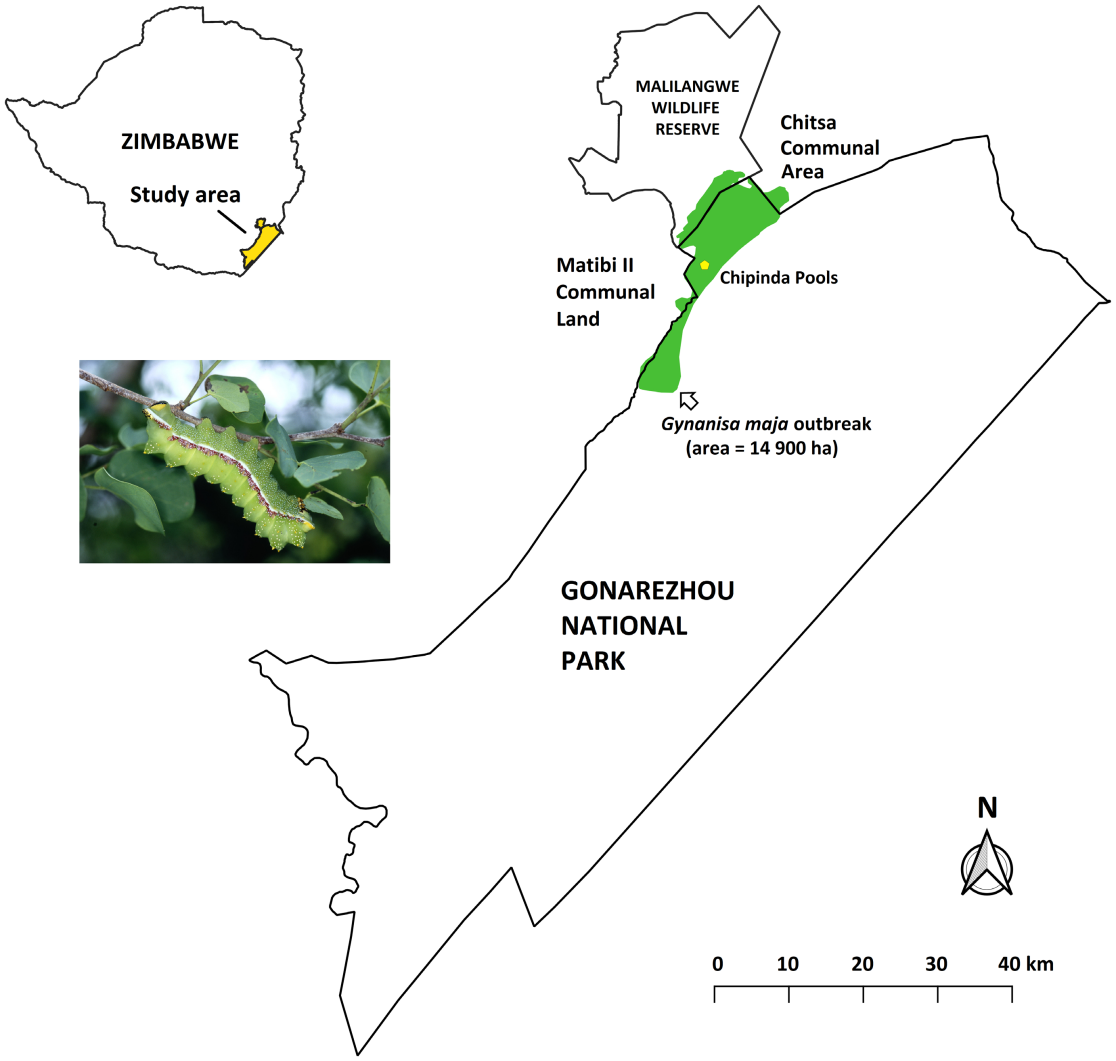
Map showing the location of the *Gynanisa maja* outbreak. Inset shows *G. maja* caterpillar on a *Colophospermum mopane* plant.

The outbreak occurred in the northern region of Gonarezhou National Park (GNP) and extended a short distance into the southern part of neighbouring Malilangwe Wildlife Reserve (MWR) (Figure 1). Rainfall in the affected area is seasonal with most precipitation occurring between November and March (mean ≈ 499 mm per annum at Chipinda Pools, which is situated within the area of the outbreak) (Gandiwa et al., 2016). The vegetation within the affected area is predominantly mopane (*C*. *mopane*) woodland on soils derived from basalt, with *Combretum imberbe*, *Philenoptera violacea*, *Dalbergia melanoxylon*, *Pterocarpus brenanii* and *Acacia nigrescens* being less common components of the woody layer (Clegg & O'Connor, 2012; Cunliffe et al., 2012).

The outbreak area was flown using a Savannah‐S fixed‐wing aircraft (ICP, Piedmont, Italy) over a single session and its boundary was digitized using a handheld Global Positioning System device. The expanse of the outbreak was easily discernable on account of the extensive defoliation of the mopane trees. This was then refined by mapping the extent of the defoliation in Quantum GIS v3.26 (QGIS Development Team, 2022) using Sentinel 2 satellite imagery and the Normalized Difference Vegetation Index (NDVI). The NDVI index is sensitive to phenological changes in vegetation cover and is used to measure greenness or the degree of green leaf cover of vegetation (Gandhi et al., 2015; Huang et al., 2021). NDVI values range from −1 to 1 with low values representing low vegetation cover and higher values corresponding to healthy vegetation (Huang et al., 2021).

Three sample plots (each measuring 25 × 5 m) were randomly located within the MWR section of the defoliated area, in areas of medium tree height (3–5 m). Each plot was thoroughly inspected, and the larvae found were collected by hand, and the number per plot was recorded. Most larvae were picked off shrubs and trees, but some were also found on the ground. A sample of 70 caterpillars (5th instar stage) were weighed, and the average mass of a caterpillar was calculated. For each plot, the live biomass of caterpillars per hectare was calculated by multiplying the average weight of a caterpillar by the number counted in the plot and dividing the result by the plot area. The mean density of caterpillar biomass for the outbreak was estimated by averaging the estimates for the three plots. This figure was then multiplied by the area of the outbreak to estimate the total caterpillar biomass.

Following the outbreak event, six sample plots (each measuring 30 × 30 m) were randomly located within the GNP section of the defoliated area for vegetation assessment. Within each plot, the species, height class (shrubs = 0–3 m, trees = >3 m) and canopy dimensions of woody plants were recorded. The degree of defoliation and subsequent foliage regrowth was visually assessed for each woody plant and scores were assigned based on a modified 9‐point scale (0, 1, 2–10, 11–25, 26–50, 51–75, 76–90, 91–95, 96–100%). Spearman's rank correlations (ρ) were used to test the relationship between plant height and the degree of defoliation or recovery (R Core Team, 2023). Informal unstructured interviews were conducted with locals to determine local knowledge of past similar *G. maja* outbreak events in the area.

## 3 RESULTS

From satellite image analysis, pixels with NDVI values 0.1 ≤ *x* ≤ 0.35 marked the defoliated area while values >0.35 represented healthy vegetation and <0.1 corresponded with cleared agricultural fields. The extent of the *G. maja* outbreak in the study area was estimated at 14,900 ha (Figure 1). A total of 1443 larvae were collected from the three plots sampled at MWR and of these, *G. belina* larvae constituted only 0.8%, the remainder being *G*. *maja*. Where both *G. maja* and *G. belina* larvae were recorded, they were found present on the same trees (i.e., no host plant exclusion by either species). The average weight of a caterpillar was 10.2 g, and the mean number of caterpillars per ha was 38,187, which equates to a biomass of 390 kg/ha (Table 1). Given a total outbreak area of 14,900 ha, the total mass of *G. maja* larvae in the affected area was estimated at 5811 tons.

**TABLE 1:** Field data on *Gynanisa maja* larvae occurrence at Malilangwe Wildlife Reserve and Gonarezhou National Park between December 2022 and January 2023.

Malilangwe Wildlife Reserve				Gonarezhou National Park			
Plot	Number of larvae picked	Number per hectare	Mass per hectare (kg)	Plot	Number of plants sampled	Mean defoliation score	Mean recovery score
1	877	70,160	716	1	33	91–95	96–100
2	333	26,640	272	2	62	11–25	96–100
3	222	17,760	181	3	63	2–10	76–90
				4	76	96–100	96–100
				5	39	91–95	96–100
				6	44	91–95	96–100

**FIGURE 2:**
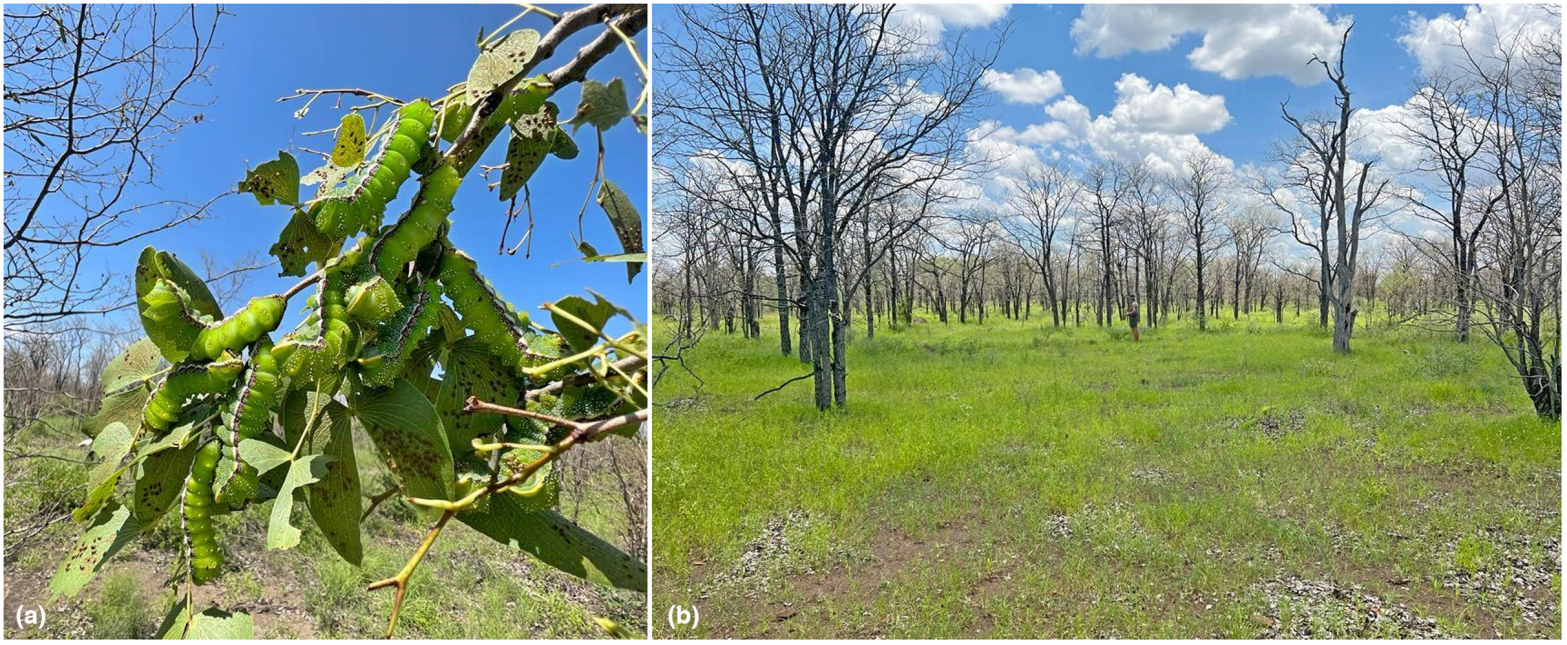
*Gynanisa maja* larvae feeding on a *Colophospermum mopane* plant (a) and an example of a defoliated stand of mopane trees (b).

**FIGURE 3:**
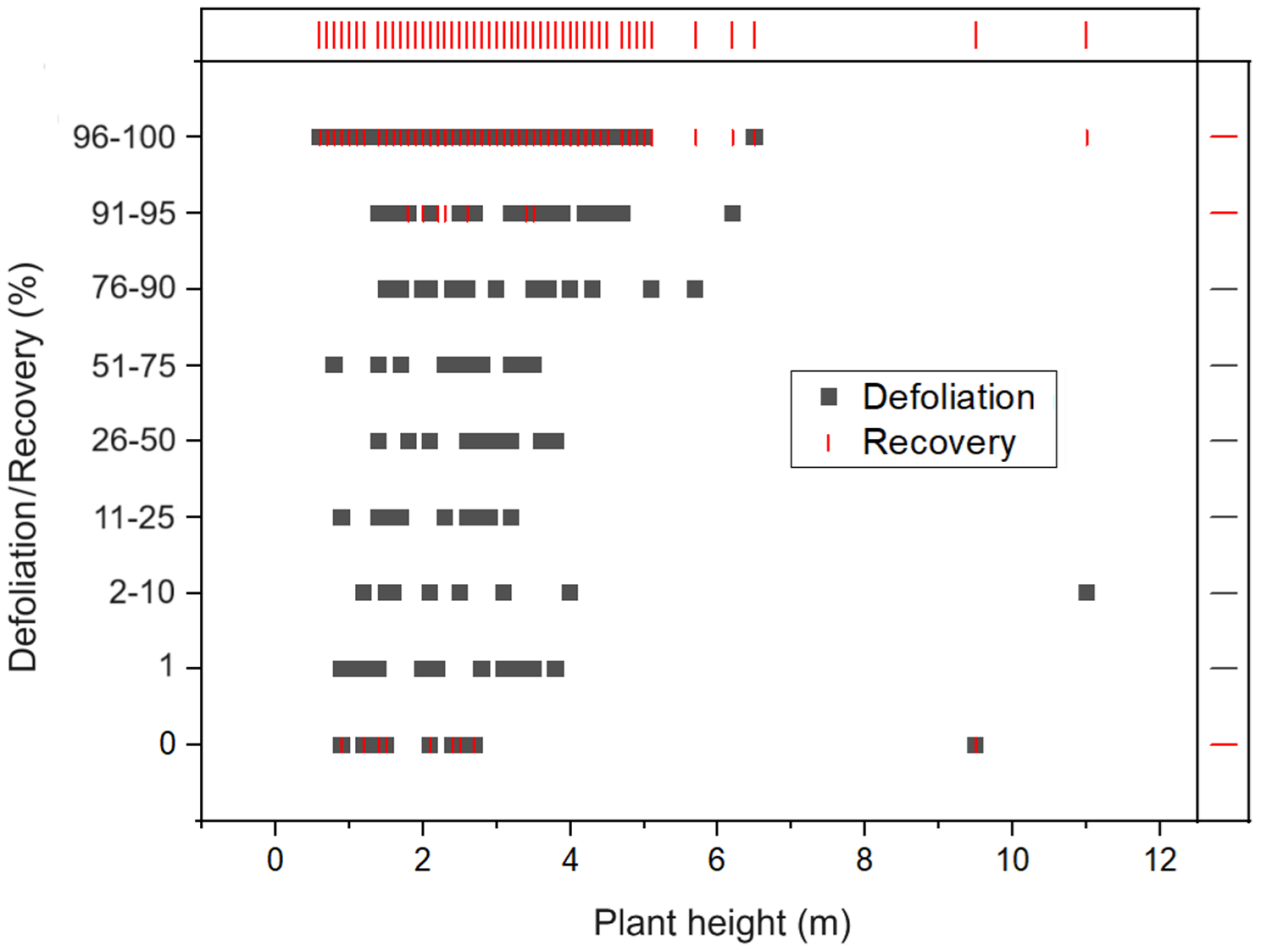
Scatter plot showing the distribution of defoliation and recovery data in relation to plant height.

A total of 10 species constituted the 317 woody plants found inside the six plots sampled at GNP with *C. mopane* making up the majority (84%) (Appendix). Within the six plots, *G. maja* larvae were only found feeding on *C. mopane* (Figure 2). The level of defoliation was high (mean rank = 76–90%, mode = 96–100%, *n* = 267) and similarly, recovery post‐defoliation was also high (mean and mode = 96–100%, *n* = 267) (Table 1, Figure 3). Plants resprouted within 21 days of defoliation. We found no relationship between plant height and the degree of defoliation (*ρ* < 0.08, df = 265, *p* = .189) or recovery (*ρ* < 0.08, df = 265, *p* = .173).

## 4 DISCUSSION

This *G. maja* outbreak was spectacular, and from interviews with locals, it appears that an event of similar magnitude was last witnessed in the 1960s. We note that while *G. belina* eruptions are common in the area, very few *G. belina* larvae were recorded during this outbreak (<1%). Either *G. maja* outcompeted *G*. *belina*, or the two species are subject to different eruption triggers. Unfortunately, the long intervals between *G*. *maja* outbreaks (≈ 60 years in this case) make it difficult to collect sufficient data to answer these questions. It should also be noted that this outbreak was not an isolated incident but rather one of several outbreaks, albeit smaller, that were reported at the same time in the surrounding communal lands.

The live weight of caterpillars per hectare was equivalent to five times the biomass density of large herbivores at MWR (68 kg/ha), and the total estimated mass of caterpillars (5811 tons) was equivalent to 3369 elephants (using 1725 kg as the average weight of an elephant following Coe et al., 1976). Considering that this represents approximately one third of GNP's estimated population of 10,800 elephants (Dunham, 2022) concentrated within 3% of the park, this represents a substantial biological event. During this outbreak, there were so many caterpillars that the sound of their feeding was clearly audible.

Larvae of *G. maja* are polyphagous and have been reported to feed on the foliage of several tree species (Sileshi et al., 2007), including *Sclerocarya birrea* which was present in the sampled plots. Despite there being several species of woody plants in the plots sampled in GNP, larvae were only found on *C. mopane*. This finding was consistent with the work of Ditlhogo et al. (1996) and Fakazi et al. (2021) on *G. belina* who showed that larvae are highly selective and almost exclusively feed on *C. mopane* except in instances where mopane trees are not available. Previous estimates of forage loss from *G. maja* could not be sourced from the literature but assuming their feeding is comparable to that of *G. belina*, then following Styles (1994), an estimated 290,550 tons of mopane leaves were consumed during the outbreak.

The effects of defoliation by *G. maja* on mopane woodlands are potentially substantial. Although *G. maja* larvae have a relatively short lifespan (6 weeks), their exceptionally large numbers during an eruption give the species an unmatched ability to rapidly defoliate large areas of woodland (Fajvan & Wood, 1996). Though our data indicate that *C. mopane* showed good regeneration after defoliation, explosion events of this nature can result in 100% loss of leaves over a short period (Hartnett et al., 2012) resulting in a temporary shortage of food for other browsers. Ditlhogo et al. (1996) found that defoliation impacted fruit production in mopane trees with 86% of defoliated plants failing to bear fruit, and shrubs being more severely impacted than trees. In contrast, we found that both shrubs and trees experienced similar levels of defoliation and that there were no differences in foliage recovery.

Defoliation can also disrupt plant community structure through competitive effects of subcanopy species on the canopy layer, which is triggered by increased light penetration to the under‐canopy layer (Carson et al., 2004; Duffy et al., 2018; Fajvan & Wood, 1996). Furthermore, outbreaks of larvae introduce food bursts in the trophic system which can lead to increased insectivore activity and altered energy flow interactions (Carson et al., 2004). Interestingly, bird activity was less than expected during the outbreak, with only hornbills (Bucerotidae) and rollers (Coraciidae) utilising the caterpillars as a source of food. The caterpillars of *G*. *maja* have a relatively tough skin and consequently, it may be difficult for other bird species to feed on them. A significant number of dead unconsumed caterpillars were found on the forest floor after the host trees had been defoliated.

Population explosions of edible insects are important events in Sub‐Saharan Africa as they have a socio‐economic benefit to the livelihoods of local communities (Bara et al., 2022; Thomas, 2013). Larvae of *G. maja* are an important source of protein and harvests can be preserved for future consumption or sold thereby enhancing food security and household incomes (Mbata & Chidumayo, 2003; Nemadodzi et al., 2023). During this outbreak, many people travelled from far and wide to the Chitsa communal area to harvest the caterpillars. Insect eruption events are poorly understood but are generally believed to be triggered by climatic factors, mainly rainfall and temperature (Koricheva et al., 2012; Spear et al., 2021). We could not associate the *G. maja* outbreak with any obvious environmental conditions and speculate that it may have been caused by subtle triggers that are not easily identified. We, therefore, encourage more research on the species and its relation to climatic and environmental conditions to enable ecological modelling and prediction of future eruption events.

## AUTHOR CONTRIBUTIONS

**Allan Tarugara:** Conceptualization (equal); formal analysis (equal); investigation (lead); methodology (equal); visualization (lead); writing – original draft (lead); writing – review and editing (equal). **Bob Mandinyenya:** Conceptualization (equal); formal analysis (equal); investigation (equal); methodology (equal); writing – review and editing (equal). **Bruce W. Clegg:** Conceptualization (equal); formal analysis (equal); investigation (equal); methodology (equal); writing – review and editing (equal).

## CONFLICT OF INTEREST STATEMENT

The authors declare no conflict of interest.

## Supporting information


Appendix S1 and S2.

## Data Availability

Data for this study has been submitted in the Dryad repository as ‘Tarugara, Allan; Mandinyenya, Bob; Clegg, Bruce (2023), An outbreak of Gynanisa maja larvae in the south‐eastern lowveld of Zimbabwe’. https://datadryad.org/stash/share/9706jpnqvrGZ45juG_rAywjtL6XItDmKzsT7Jl15AYs.
